# Minutes of the First Annual Meeting of the Ohio State Dental Association

**Published:** 1867-02

**Authors:** Will. Taft


### Proceedings of Societies.

### MINUTES OF THE FIRST ANNUAL MEETING OF THE OHIO STATE DENTAL ASSOCIATION.

The first annual meeting of the Ohio State Dental Society met pursuant to adjournment on Tuesday, January 15, 1867, in the city of Columbus, at 10 o'clock, A. M.

Dr. George Watt, the President, being absent on account of severe illness, Vice-President George W. Keely took the chair.

Members present: Drs. George W. Keely, W. P. Horton, L. Buffett, J. Taft, N. W. Williams, A. A. Blount, A. W. Maxwell, C. M. Kelsey, M. De Camp, C. H. Harroun, W. E. Dunn, H. Newington, F. H. Rehwinkel, A. R. Lord, J. M. Rhodes, C. S. Cady, J. W. Wortman, J. Williams, T. W. French.

Dr. W. P. Horton was appointed Secretary *pro tem.*

Minutes of the first semi-annual meeting read and approved.

The reports of committees, now in order, were deferred on account of absentees. The President, on motion of the Society, filled the committees *pro tem*, as follows:

*Executive Committee.*—Drs. Kelsey, J. Taft, A. W. Maxwell.

*Committee on Membership.*—Drs. F. H. Rehwinkel, M. De Camp, J. B. Beauman.

*Committee on Ethics.*—Drs. J. Taft, W. E. Dunn, C. S. Cady.

Dr. J. Taft, Chairman of the committee on Ethics, remarked that they had no special report to make at this time. So far as the committee are aware, all the members of the Society have conformed to the spirit of the Code.

On motion, a committee of three to nominate officers for the ensuing year was appointed, consisting of Drs. C. M. Kelsey, L. Buffett and M. De Camp.

A recess was then taken until the Committee on Membership was ready to make their report.

Dr. F. H. Rehwinkel, Chairman of the Committee on Membership, offered the following:

Your Committee on Membership respectfully report the names of the following gentlemen as eligible, worthy and well qualified to become members of this Association, viz.: Drs. D. R. Jennings, Ravenna; Will. Taft and A. Berry, Cincinnati; W. M. Herriott and Wm. M. Chappelear, Zanesville; J. L. Dunlap, Chillicothe; J. C. Whinery, Salem; H. M. Edson, Mt. Vernon; W. R. Lilly, Circleville; F. Emmons, Delaware.

F. H. REHWINKEL,<br>M. DECAMP,<br>J. B. BEAUMAN, } *Com.*

The report was accepted, and the gentlemen named were all unanimously elected members.

TREASURER'S REPORT.

*To the Officers and Members Ohio State Dental Society:*

Your Treasurer would respectfully report that at the meeting of the Association on the 27th of June last, he received funds as follows:

| | | |
|---|---|---|
| From the Secretary | $93 00 | |
| Members | 27 00 | |
| Total | $120 00 | |
| Paid Dr. J. Taft on account | | $16 00 |
| " H. A. Smith to purchase Secretary's book, | | 8 00 |
| " J. Taft for reporter | | 35 00 |
| " J. B. Beauman for use of hall | | 10 00 |
| " for Treasurer's book | | 88 |
| | | $69 88 |
| Balance in Treasury | $50 12 | |

All of which is respectfully submitted.

M. DECAMP, *Treasurer.*

Report accepted and referred to an Auditing Committee.

On motion, the annual dues were fixed at three dollars. ($3 00.)

The Auditing Committee would respectfully report that they have carefully examined the Treasurer's Report, and find the same correct.

WILL TAFT,<br>C. M. KELSEY, } *Com.*

The Committee on Nominations reported the following names as officers for the ensuing year:

President—George Watt, Cincinnati; First Vice-President—George W. Keely, Oxford; Second Vice President—W. P. Horton, Cleveland; Recording Secretary—Will. Taft, Cincinnati; Corresponding Secretary—A. W. Maxwell, Galion; Treasurer—A. Berry, Cincinnati.

On motion, the report was accepted.

The Association then went into an election, and the candidates above nominated were elected.

On motion, the Secretary was authorized to send a dispatch to Dr. George Watt, informing him of his re-election, and the regrets of the Society at his inability to be present and preside at this, the first annual meeting of the Society.

Adjourned to meet at 2 P. M.

### AFTERNOON SESSION.

The Association was called to order at the hour appointed, Vice-President Keely in the Chair. The minutes of the morning session were read and approved.

The reading of essays was next in order, and called for; but there being none in preparation, the order was passed.

The first subject for discussion, Anæsthesia, was then taken up, and elicited much interest. All the agents and modes of administration were taken up and fully debated.

There seemed to be no general preference for any particular agent.

Adjourned until to-morrow morning, at 9 o'clock.

MORNING SESSION, JANUARY 16.

Meeting called to order. Minutes read and approved.

On motion, the discussion on Anæsthesia was closed, and the subject next in order, the bill to regulate the practice of Dentistry in the State of Ohio, was taken up. The discussion was spirited, and manifested much earnestness; showing the members to be fully awake to the importance of the measure. The debate was mainly upon the ten year restriction clause. The sentiment was generally against the clause as depriving the bill of its greater merit. During the discussion, Dr. Rhodes read an essay, which on motion, was referred to the Publication Committee.

On motion, the following gentlemen were appointed a committee to confer with the Legislature in regard to the passage of the bill, and to take charge of all matters pertaining thereto: Drs. J. Taft, A. A. Blount, W. P. Horton, J. B. Beauman and J. M. Rhodes.

On motion, the local societies throughout the State were recommended to send representatives to Columbus to act in concert with the above committee.

The undersigned would give notice that an important amendment to the Constitution will be proposed by them, which will change Sec. 2d so as to read: "Active members must be twenty-one years of age, and be regular and honorable practitioners of Dentistry in the State of Ohio."

F. H. Rehwinkel,
J. B. Beauman,
M. DeCamp.

On motion, the "Rubber Question" was made the special order of discussion for the Afternoon Session.

Adjourned until 1½ P. M.

AFTERNOON SESSION.

President Keely in the Chair.

Prof. J. Taft moved that a committee of two be appointed to present instruments and appliances to the Society. Carried. Drs. C. H. Harroun, of Toledo, and W. R. Lilly, of Circleville, were appointed said committee.

On motion, the first hour of the session to-morrow morning be devoted to such presentation.

The "rubber question" was taken up, and occupied the remainder of the afternoon.

Adjourned to meet at 7 P. M.

EVENING SESSION.

Minutes of the morning session read and approved.

The discussion on the "rubber question" was then resumed.

Dr. J. Taft laid before the Association the result of his trip to New York. This will probably be placed before the profession in full at another time.

Dr. Keely was called upon to give some of his experience upon the somewhat extensive tour he took in relation to the "rubber interest." to which he responded in a very happy manner.

Dr. J. Taft presented the following resolutions, which were adopted:

*Whereas*, Dr. I. J. Wetherbee, of Boston, Mass., has, in our opinion, done all in his power to resist the unjust and extortionate demands of the "Goodyear Dental Vulcanite Co.," thereby involving himself to a serious extent; therefore, be it

*Resolved*, That the whole Dental Profession is under deep obligations to Dr. I. J. Wetherbee for the effort he put forth in contesting the suit brought against him by the Dental Vulcanite Co.

*Resolved*, That we raise funds to the amount of $800, (eight hundred dollars,) to relieve Dr. Wetherbee of the financial burden occasioned by the recent trial.

On motion, a vote of thanks was tendered to Dr. George W. Keely for his services in collecting funds for the Protective Association.

On motion, the names of the members of the Association were called, giving each one the opportunity to state the amount he was willing to contribute to the Wetherbee Fund.

On motion, Dr. J. Taft was appointed to take charge of the above fund.

Adjourned until to-morrow morning, at 9 o'clock.

### THURSDAY MORNING, JANUARY 17.

Association called to order. Minutes read and approved.

On motion, Dr. Rehwinkel was added to the Committee on Instruments and Appliances.

The Report of the said Committee was then taken up:

*Gentlemen:*—Your Committee on Instruments and Appliances beg leave to present to you the following list of instruments placed in their hands for examination:

One Automatic Plugger, invented and manufactured by Dr. W. G. Redman, of Louisville, Ky. This instrument shows the skill of an ingenious mind, and is a very handsome instrument; and would be an ornament to the table of any operator.

One Automatic Plugger, from Dr. Poor, of Dubuque, Iowa. This is the most simple and easily used—giving almost, if not quite, the peculiar blow of the mallet itself.

One Automatic Plugger, invented by Dr. George F. Foote, of New York city. This instrument is so well described in the *Dental Cosmos* as to need no comment from us.

A lot of instruments and appliances presented by Dr. S. D. Palmer, and manufactured by S. S. White, of Philadelphia. Among these are a set of Rubber Buff Wheels, for polishing rubber plates.

An Articulator, made of brass, and double jointed, giving all the movements necessary for obtaining and maintaining correct articulations.

A set of one dozen Chisels, from patterns furnished by Dr. Darby. These are doubtless very useful instruments.

A set of Pluggers, from patterns devised by Dr. Isaiah Forbes, of St. Louis, Mo. The merit claimed for these consists in the peculiar curvature of the working ends. The serrations are well defined, and, altogether, seem to be very desirable instruments.

A set of Dr. C. Palmer's Nerve Instruments. These are familiar to every progressive Dentist, and so highly approved by their almost universal use, that we deem it unnecessary for us to dwell upon them further.

The last upon our list is a Speculum and Duct Compressor. This is one of the most valuable improvements we have ever seen, and answers the purpose for which it is intended admirably. This instrument once upon the operator's table will ever be considered a most faithful accessory. Dr. E. Bradley, of Dayton, Ohio, is the inventor of the above instrument.

C. H. Harroun,<br>

W. R. Lilly,<br>

F. H. Rehwinkel, } *Com.*

By a unanimous request of the Society, Dr. C. H. Harroun gave a most excellent description of his method of artificial restoration of the palatal fissures. Dr. Harroun has evidently devoted much time and profound study to this specialty of Dental Science; not studying, to copy from others, but originality in his appliance. From his effort, we have a far simpler appliance than any yet before the profession. The Doctor's effort and success has placed him in a position not inferior to that of the discoverer.

Dr. W. M. Herriott, of Zanesville, presented to the Association a case in which he supplied an artificial nose, upper lip, and nearly all of the superior teeth. The operation was certainly a remarkable one, and it is hoped that Dr. Herriott will lay the case before the profession in detail.

Bills presented by Drs. J. Taft, A. W. Maxwell, and W. P. Horton, were approved, and ordered paid.

The following was offered by the Committee on Membership :

The Committee on Membership beg leave respectfully to report, that in addition to the names of the gentlemen recommended for membership, there are several applications of gentlemen who are not, and were not present themselves. We recommend that these cases lay over until our next meeting, when they must be present in person for examination.

There are several applications of gentlemen for membership who are not at this time eligible; and upon recommendation of your Committee, they were declared honored guests of this Society, and all courtesies extended to them. No further applications having been presented to the Committee, they beg leave to be discharged.

M. DeCamp,<br>J. B. Beauman,<br>F. H. Rehwinkel, } *Com.*

Accepted and the Committee discharged.

On motion, Dental Education and Dental Ethics were made the special order for the fore part of the afternoon session.

Adjourned until 1½ P. M.

AFTERNOON SESSION.

Association called to order. Minutes read and approved.

On motion, it was

*Resolved*, That the next semi-annual meeting of the Society be held in Columbus on the first Tuesday of June next.

On motion, the Code of Ethics as adopted by the American Dental Association was substituted for the one formerly adopted by this Society.

It was resolved to have the Code stereotyped, with such other matter as Prof. J. Taft (into whose hands the duties of the work were placed), might deem advisable; and that the Association defray the expenses so incurred.

On motion, the regular order of business was suspended, and miscellaneous business and discussions announced in order.

The bill for use of hall was presented, approved, and ordered paid.

The Society resolved to make a donation of ten dollars to the Young Men's Christian Association of this city.

The President announced the following Committees for the ensuing year:

*Executive Committee.*—Drs. L. Buffett, W. M. Herriott, J. B. Beauman.

*Committee on Membership.*—Drs. F. H. Rehwinkel, M. DeCamp, B. T. Spelman.

*Publication Committee.*—Drs. Will. Taft, H. A. Smith, N. W. Williams.

*Dental Ethics.*—Drs. J. Taft, J. W. Rhodes, F. H. Rhewinkel.

The discussions of the afternoon were of a social rather than a professional character. Individual office experiences were given, and created much amusement; and thus the afternoon session formed a very pleasant ending to a pleasant session.

Adjourned to meet in Columbus on the first Tuesday of June next.

WILL. TAFT, *Secretary.*

